# Nodal induces apoptosis and inhibits proliferation in ovarian endometriosis-clear cell carcinoma lesions

**DOI:** 10.1186/s12885-019-5539-y

**Published:** 2019-04-03

**Authors:** Rinako Miura, Ako Yokoi, Toshihide Matsumoto, Yasuko Oguri, Miki Hashimura, Masataka Tochimoto, Sabine Kajita, Makoto Saegusa

**Affiliations:** Department of Pathology, Kitasato University School of Medicine, 1-15-1 Kitasato, Minami-ku, Sagamihara, Kanagawa 252-0374 Japan

**Keywords:** Nodal, Endometriosis, Ovarian clear cell carcinoma, TGF-β, GSK-3β, Smad, Apoptosis, Cell proliferation

## Abstract

**Background:**

Expression of Nodal, a member of the TGF-β superfamily, is commonly absent in differentiated tissues, while its re-expression occurs in a variety of human malignancy. However, little is known about its involvement in ovarian tumorigenesis. Herein, we focused on the functional roles of Nodal in ovarian endometriosis-carcinoma lesions.

**Methods:**

Regulation and function of Nodal and its associated molecules, including Smad2, GSK-3β, and several cell kinetics-related molecules, were assessed using clinical samples consisting of 108 ovarian carcinomas and 33 endometriotic lesions, as well as ES-2 (ovarian clear cell carcinoma; OCCCa) and Ishikawa (endometrial carcinoma) cell lines.

**Results:**

Nodal expression was significantly higher in endometriosis and OCCCa lesions as compared to that of non-OCCCas, with positive correlations to phosphorylated forms of both Smad2 (pSmad2) and GSK-3β. When compared to endometriotic lesions, the expression of Nodal and pSmad2 was significantly decreased in OCCCa. Treatment of Ishikawa cells with TGF-β1 resulted in transcriptional upregulation of Nodal, along with increased pSmad2 expression, while inhibition of GSK-3β also induced an increase in Nodal expression at the posttranslational level. Both ES-2 and Ishikawa cells stably overexpressing Nodal had increased susceptibility to apoptosis in response to treatment with cisplatin and doxorubicin, respectively, together with higher cleaved caspase-3 expression and decreased Bcl2/Bax ratio. Moreover, the stable Nodal-overexpressing cells showed reduced cell proliferation, along with increased expression of p27^kip1^ and p21^waf1^. In clinical samples, a significantly higher number of apoptotic cells and lower Ki-67 labeling indices were observed in Nodal-positive as compared to Nodal-negative OCCCa.

**Conclusions:**

These findings suggest that Nodal is a multifunctional cytokine involved in the modulation of cell kinetics in ovarian endometriosis-OCCCa lesions.

**Electronic supplementary material:**

The online version of this article (10.1186/s12885-019-5539-y) contains supplementary material, which is available to authorized users.

## Background

Ovarian epithelial carcinoma (OECa), which consists of four histologically distinct subtypes, including serous (OSeCa), mucinous (OMuCa), endometrioid (OEmCa), and clear cell (OCCCa) types, has the worst prognosis of all gynecological malignancies [1]. Of these, OCCCa is not just another type of OECa, but a distinct entity with a specific etiology and biological composition [2]. For example, the most effective treatment for OECa is platinum-based chemotherapy, such as cisplatin (CDDP), while OCCCa often shows chemoresistance and the clinical outcome in its advanced stage is generally unfavorable, despite their slow growth [3–5].

Endometriosis, the presence of ectopic endometrial tissue, is a common gynecological disease, with an estimated prevalence of about 10% in women of reproductive age and is thought to be a precursor of both OCCCa and OEmCa [6–8]. A high concentration of free iron due to repeated hemorrhage and inflammation is frequently detected in ovarian endometriotic cysts, and promotes development of the tumors through iron-induced persistent oxidative stress and DNA damage [9]. Interestingly, such an inflammatory reaction also alters the expression of transforming growth factor β (TGF-β), which can play multiple roles in human tumorigenesis by behaving as a tumor suppressor at early stages and a tumor promoter at late stages [10–13].

Nodal, a member of the TGF-β superfamily, plays vital roles in differentiation of the endoderm and mesoderm during embryogenesis [14–16]. Nodal expression is commonly absent in differentiated tissues [17], while its re-expression occurs in a variety of human malignancies. However, published data regarding its functional roles in tumor development and progression are conflicting. For example, inhibition of Nodal signaling reduces cell invasiveness, colony formation, and tumorigenicity in melanomas [18], while overexpression of Nodal induces a decrease in the number of metabolically active cells in OECa [19]. Thus, the role of Nodal may be dependent on the tumor cellular microenvironment and associated cell type.

In this study, we found significant upregulation of Nodal expression in ovarian endometriosis-OCCCa lesions, when compared with other OECa histological subtypes. We further elucidated an association between TGF-β/Smad signaling and the functional role of Nodal with regard to the rate of tumor cell apoptosis and proliferation in the OCCCa lesions.

## Methods

### Clinical cases

A total of 108 cases of OECa including 57 OCCCas, 31 OEmCas, 10 OSeCas, and 10 OMuCas, surgically resected at Kitasato University Hospital in the period from 2000 to 2017, were selected from our patient records, according to the criteria of the 2014 World Health Organization classification [20]. Endometriotic lesions adjacent to carcinomas were also investigated in 17 OCCCa and 14 OEmCa cases, as well as 33 samples of endometriotic lesions without carcinomas. The mean age of the patients was 56.1 years (range from 29 to 81 years). Fifty cases were subcategorized as stage I and 38 as stage II to IV, according to the criteria of the International Federation of Gynecology and Obstetrics (FIGO) [21]. None of the patients had undergone chemotherapy or radiation therapy before surgery. All tissues were routinely fixed in 10% formalin and processed for embedding in paraffin wax. In addition, 41 fresh OECa tissue samples (11 OCCCas, 11 OEmCas, 11 OMuCas, and 8 OSeCas) were used for RT-PCR (reverse transcription-polymerase chain reaction) and western blot assays. Approval for this study was given by the Ethics Committee of the Kitasato University School of Medicine (B17–275).

### Antibodies and reagents

Anti-Nodal and anti-β-actin antibodies as well as CDDP, doxorubicin (Dox), MG132, and lithium chloride (LiCl) were purchased from Sigma-Aldrich Chemicals (St. Louis, MO, USA). Anti-X-linked inhibitor of apoptosis (XIAP), anti-Bax, anti-Glycogen Synthase Kinase (GSK)-3β, and anti-p27^Kip1^ antibodies were bought from BD Biosciences (San Jose, CA, USA). Anti-p21^waf1^, anti-Cyclin D1, anti-Bcl2, and anti-Ki-67 antibodies were purchased from Dako (Copenhagen, Denmark). Anti-cleaved caspase-3 and anti-phospho(p) GSK-3β at Ser9 (pGSK-3β) antibodies were obtained from Cell Signaling Technology (Danvers, MA, USA). Anti-Smad2 and anti-pSmad2 at Serine 255 (pSmad2) antibodies were from Abcam (Cambridge, MA, USA). Anti-Cyclin A2 antibody was from Novocastra (Newcastle, UK). Recombinant transforming growth factor (TGF)-β1 was purchased from R&D Systems (Minneapolis, MN, USA).

### Immunohistochemistry (IHC)

IHC was performed using a combination of the microwave-oven heating and Histofine Simple Stain MAX-PO (MULTI) (Nichirei Biosciences, Tokyo, Japan) methods as described previously [22, 23]. For evaluation of IHC findings, scoring of nuclear or cytoplasmic immunoreactivity for Nodal, pSmad2, pGSK-3β, XIAP, Bcl2, and Bax and labeling indices (LIs) of nuclear Ki-67 immunopositivity were performed as described previously [22, 23].

### In situ hybridization (ISH)

Riboprobe for TGF-β1 containing nucleotides 905 to 1479 of the *TGF-β1* gene was generated by in vitro transcription, and ISH assays were performed using the GenPoint Tyramide Signal Amplification System (Dako), as described previously [22, 23]. The ISH signal score was determined on the basis of the percentage of ISH signal-positive cells and the ISH signal intensity as described previously [22, 23].

### Apoptosis assay

Apoptotic cells were identified in HE-stained sections, according to the criteria of Kerr et al. [24]. A total of 20 fields were randomly selected, and the amount of apoptotic cells was calculated by counting the mean number of apoptotic bodies per 5 high-power fields (HPFs) as described previously [22, 23]. Areas of severe inflammatory cell infiltration and necrosis were excluded, because of the presence of uncertain cells in such lesions.

A TUNEL assay for the detection of apoptotic cells was also conducted using the In Situ Cell Death Detection kit (Roche, Tokyo, Japan), according to the manufacturer’s instructions. The number of positive cells was also analyzed by counting the mean number of TUNEL-positive cells per 10 HPFs as described previously [22].

### Plasmids

Full-length cDNA for human Nodal (Open Biosystems, Huntsville, AL, USA) was subcloned into pcDNA3.1 (Invitrogen, Carlsbad, CA, USA). The human *Nodal* promoter between − 1522 and + 23 bp (where + 1 represents the transcription start site) was amplified by polymerase chain reaction (PCR) and was subcloned into the pGL-3B vector (Promega, Madison, WT, USA). Site-mutagenesis of the putative GSK-3β phosphorylation motif in the Nodal protein (pcDNA3.1-Nodal-2TA) was also carried out by PCR using the pcDNA3.1-Nodal expression plasmid as template DNA and specific Mut-2TA primers. The identity of all constructs was confirmed by sequencing prior to use. The sequences of PCR primers employed in this study are listed in Table 1. pcDNA3.1-Smad2, pGL3B-(− 2338) p21^waf1^ luc, pGL3B-(− 1565) p27^kip1^ luc, pGL3B-(− 1467) Cyclin A2 luc, and pGL3B-(− 432) XIAP luc were used as described previously [22, 23].

**Table 1:** Primer sequences used in this study

Gene			Sequence
Nodal	Promoter	Forward	5′-ACAGCCTCCTCCCCTAAGACAGA-3’
		Reverse	5′-GAAAGCAGCACCTCCAGCCCTTA-3’
	cDNA	Forward	5′-ACCATGCACGCCCACTGCCTG-3’
		Reverse	5′-ATGTCATCAGAGGCACCCACA-3’
	mRNA	Forward	5′-AGACATCATCCGCAGCCTAC-3’
		Reverse	5′-GTCCATCTGAAACCGCTCTA-3’
	Mut-2TA	Forward	5′-GTGCTCCTTATGCTCTACTCCAACCTCTCG-3’
		Reverse	5′-ATTGGCGGCAGGCGGTGCGG-3’
TGF-β1	mRNA	Forward	5′-TACTACGCCAAGGAGGTCAC-3’
		Reverse	5′-ATTTCCCCTCCACGGCTCAA-3’
	ISH	Forward	5′-AGTTGTGCGGCAGTGGTTGA-3’
		Reverse	5′-CGGGACCTCAGCTGCACTT-3’

*Mut-2TA* mutation in GSK-3β phosphorylation motif, *ISH* in situ hybridization

### Cell lines

ES-2 (OCCCa) (Cat#CRL-1978) and TOV-21G cell lines (OCCCa) (Cat#CRL-11730) were obtained from the American Type Culture Collection (Manassas, VA, USA), and OVISE (OCCCa) (Cat#JCRB1043) and Ishikawa cell lines (EmCa)(Cat#JCRB1505) were from the National Institute of Biomedical Innovation (Osaka, Japan). The Nodal expression plasmid or empty vector was transfected into ES-2 and Ishikawa cells, and the stable overexpressing clones were established as described previously [22, 23]. Cell lines were tested annually for mycoplasma (last test date: 3/2016, negative) and all experiments were completed afterwards.

### Transfection

Transfection was carried out using LipofectAMINE PLUS (Invitrogen) as described previously [22, 23]. Luciferase activity was assayed as described previously [22, 23].

### RT-PCR

cDNA was synthesized from 2 μg of total RNA. Amplification was carried out in the exponential phase to allow comparison among cDNAs synthesized from identical reactions, using specific primers (Table 1). Primers for the *GAPDH* gene were also applied as described previously [22, 23].

### Western blot assays

Total cellular proteins were isolated and western blot assay was performed as described previously [22, 23]. The intensity of individual signals was measured using ImageJ software version 1.41 (NIH, Bethesda, MD, USA).

### Flow cytometry

Cells were fixed using 70% alcohol and stained with propidium iodide (Sigma-Aldrich) for cell cycle analysis. Cells were then analyzed by flow cytometry using BD FACS Calibur (BD Biosciences) and CellQuest Pro software (BD Biosciences).

### Statistics

Comparative data were analyzed using the Mann-Whitney *U*-test and the Spearman’s correlation coefficient, whichever was appropriate. The cutoff for statistical significance was set as *p* < 0.05.

## Results

### Nodal expression in OECas and endometriosis

**Fig. 1:**
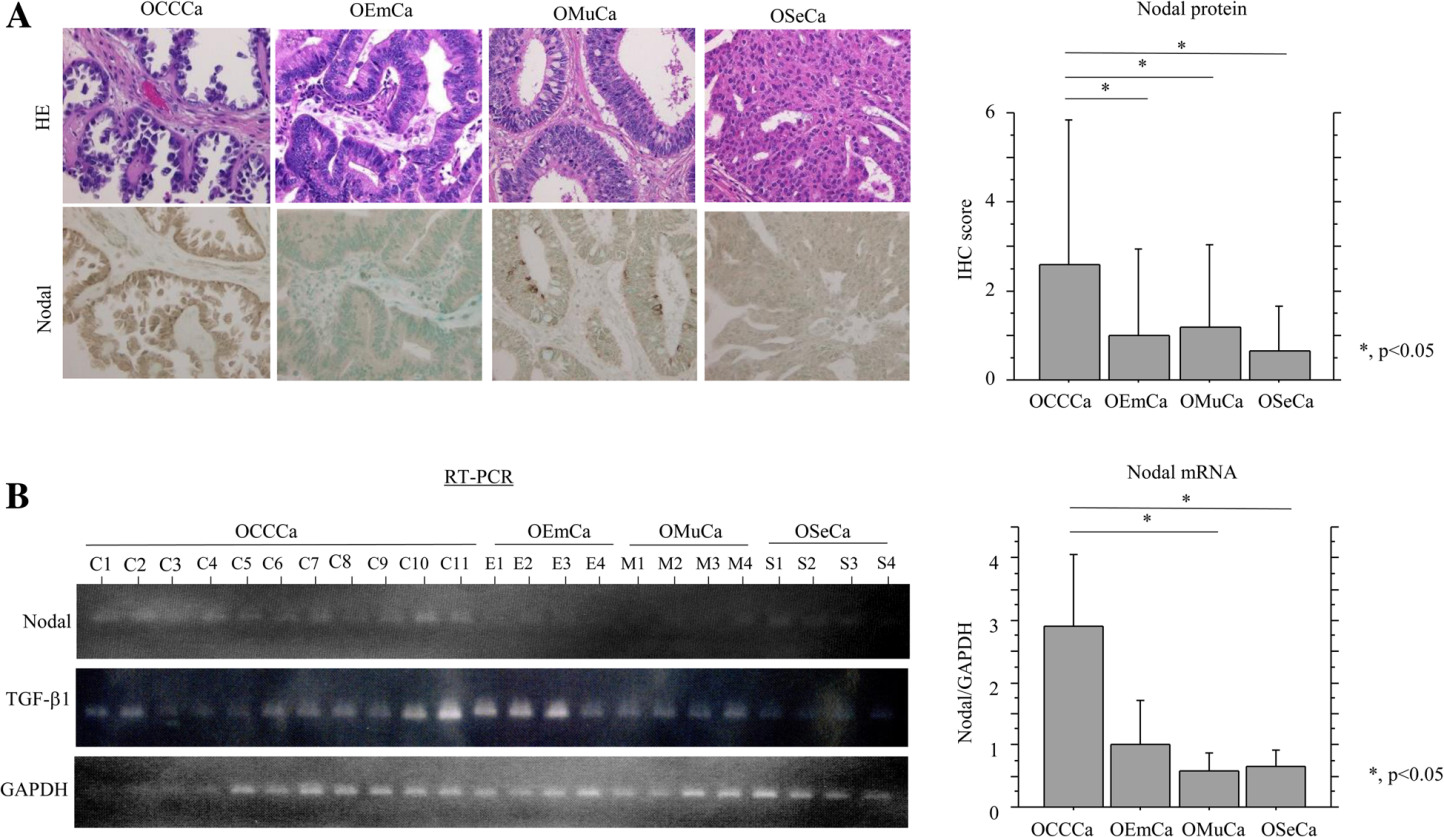
IHC and RT-PCR findings in OECas. **a** Left: staining by hematoxylin and eosin (HE) and IHC for Nodal in OECas. Note the immunopositivity for Nodal in OCCCa, in contrast to the weak or absent staining in non-OCCCas. Original magnification, × 200. Right: IHC score for Nodal in OECa. **b** Left: mRNA expression of Nodal and TGF-β1 in OECa by RT-PCR assay. Right: relative mRNA levels of endogenous Nodal in OECa were calculated by normalization to GAPDH using the NIH ImageJ program. The data shown are means ± SDs

Cytoplasmic Nodal immunoreactivity with or without nuclear staining was frequently observed in OCCCa, while sporadic distribution or absence of Nodal-immunopositive cells was observed in non-OCCCa lesions including OEmCa, OMuCa, and OSeCa. Average IHC scores for Nodal were significantly higher in OCCCa than those of non-OCCCas (Fig. 1a). Similar findings were also observed for Nodal mRNA expression, independent of TGF-β1 mRNA status (Fig. 1b).

**Fig. 2:**
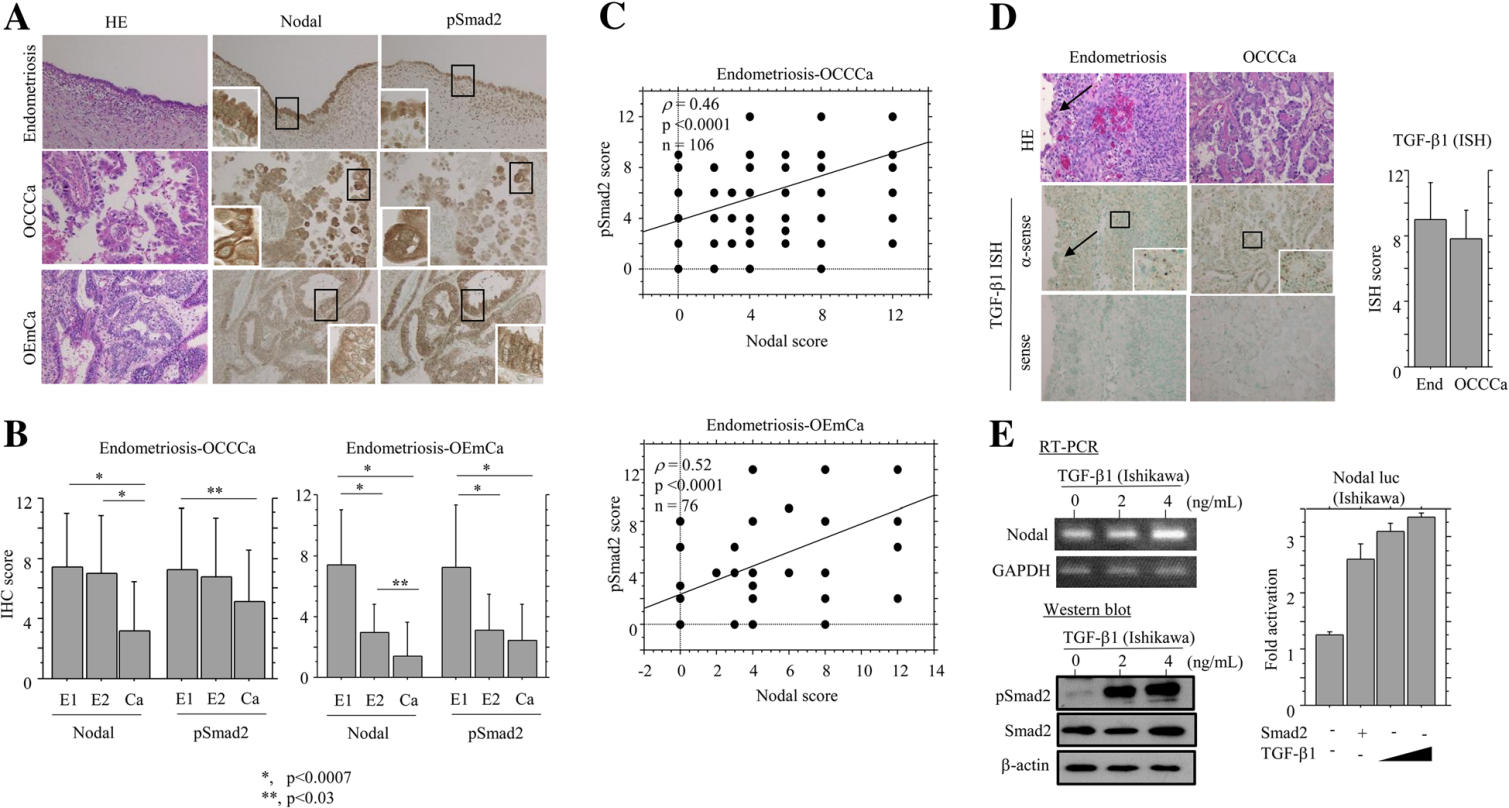
Relationship of Nodal with pSmad2 in endometriosis-OCCCa/OEmCas. **a** Staining by hematoxylin and eosin (HE) and IHC for Nodal and pSmad2 in endometriosis, OCCCa, and OEmCa. Insets show the magnified views of the boxed areas. Original magnification, × 200, × 400 (inset). **b** IHC scores for Nodal and pSmad2 in endometriosis-OCCCa (left) and endometriosis-OEmCa (right). The data shown are means ± SDs. E1, endometriosis without carcinoma; E2, endometriosis with carcinoma; Ca, carcinoma. **c** Correlation between Nodal and pSmad2 scores in a combination of endometriosis and OCCCa (upper) or OEmCa (lower). **d** Left: staining by HE and ISH for TGF-β1 mRNA in endometriosis and OCCCa. Note the strong ISH signals in stromal components of endometriosis and OCCCa cells, in contrast to a lack of the signal in the epithelial element (indicated by arrow) in the former. Insets show the magnified views of the boxed areas. Original magnification, × 100 and × 400 (inset). Right: ISH scores for TGF-β1 in endometriosis (End) and OCCCa. **e** Left: RT-PCR (left upper) and western blot analyses (left lower) for the indicated molecules in Ishikawa cells following treatment with 0, 2, and 4 ng/mL TGF-β1 for 24 h. Right: Ishikawa cells were transfected with Nodal reporter constructs, together with cotransfection of Smad2 or treatment of 2 and 4 ng/mL TGF-β1 for 24 h. Relative activity was determined based on arbitrary light units of luciferase activity normalized to pRL-TK activity. The activities of the reporter plus the effector relative to that of the reporter plus empty vector are shown as means ± SDs. The experiment was performed in duplicate

Next, we examined the association of Nodal expression with the TGF-β/Smad2 signal pathway in endometriosis and OCCCa/OEmCa. pSmad2 immunoreactivity was mainly located in cytoplasmic compartments with or without nuclear staining in OCCCa and OEmCa, while its expression was observed in both epithelial and stromal elements in endometriosis (Fig. 2a). Average IHC scores for Nodal and pSmad2 were significantly decreased in OCCCa as compared to those of both endometriotic lesions without and with carcinoma lesions (E1 and E2, respectively), while these scores showed stepwise decreases from E1 and E2 through to OEmCa (Fig. 2b). In addition, Nodal score was positively correlated with pSmad2 score in both endometriosis-OCCCa and endometriosis-OEmCa lesions (Fig. 2c).

In 10 endometriosis and 10 OCCCa cases that were investigated, positive TGF-β1 mRNA signals, as detected by ISH assay, were frequently observed in the stromal components in both endometriotic lesions and OCCCa cells, with no difference in the ISH scores between the two (Fig. 2d). Treatment with TGF-β1 induced robust dose-dependent increases in expression of Nodal mRNA and pSmad2 protein in Ishikawa cells, in contrast to the weak or absent increases observed in ES-2, OVISE, and TOV-21G cells (Additional file 1: Figure S1A). Transfection of Smad2 or TGF-β1 treatment also resulted in increased *Nodal* promoter activity (Fig. 2e). These findings suggest that Nodal expression is closely associated with TGF-β1/Smad2 status in endometriosis-OCCCa, as well as OEmCa.

### Association between Nodal expression and GSK-3β status in endometriosis- OCCCa

**Fig. 3:**
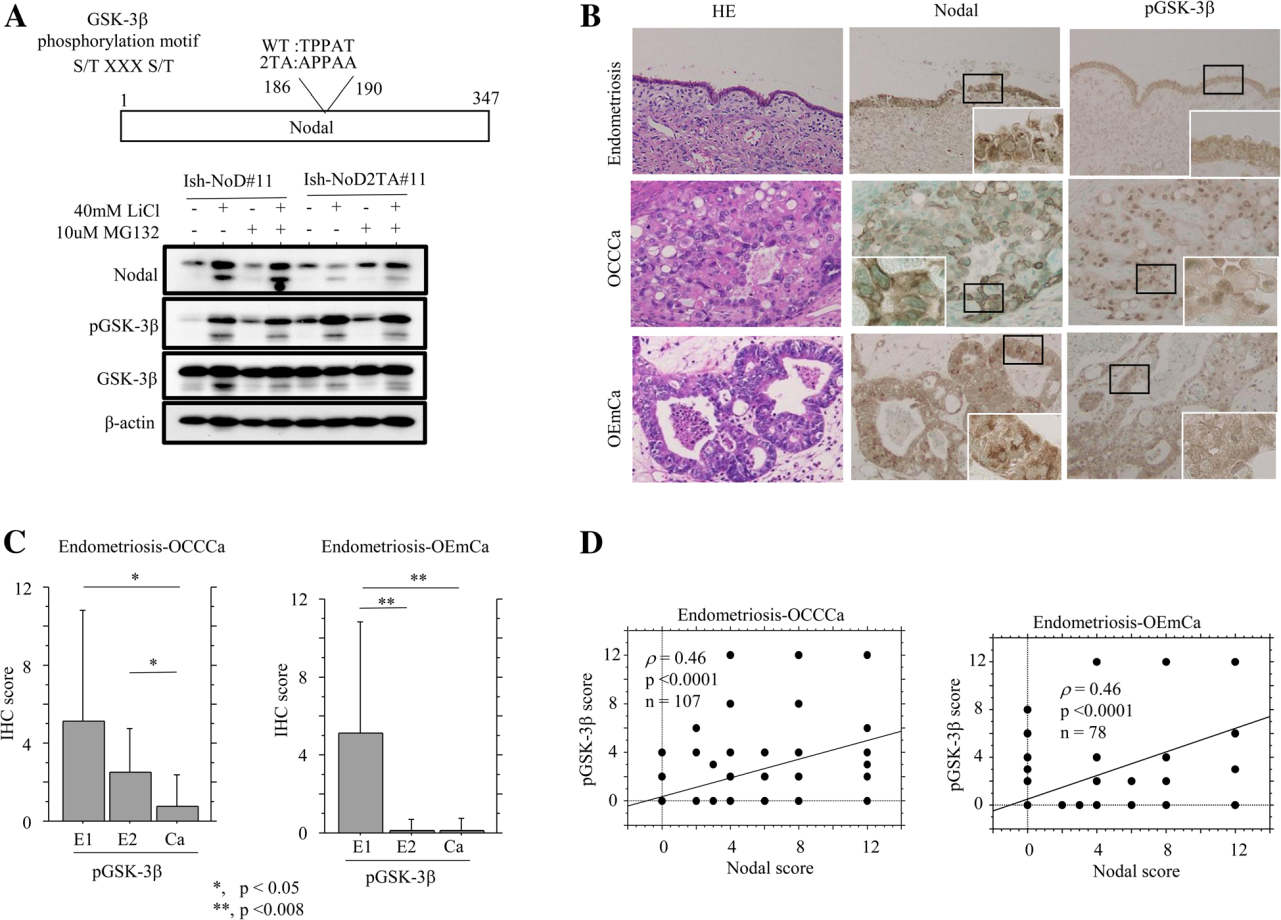
Relationship of Nodal with GSK-3β in endometriosis-OCCCa /OEmCa. **a** Upper: structure of Nodal protein showing the position of a putative GSK-3β phosphorylation motif. The corresponding constructs that were used in this study are also shown. WT, wild-type; 2TA, mutant type. Lower: western blot analysis for the indicated proteins after treatment of Ishikawa cells stably overexpressing wild-type and mutant type 2TA Nodal with 40 mM LiCl for 24 h with or without 10 μM MG132 for 5 h. **b** Staining by hematoxylin and eosin (HE) and IHC for Nodal and pGSK-3β in endometriosis, OCCCa, and OEmCa. Insets show the magnified views of the boxed areas. Original magnification, × 100 and × 400 (inset). **c**. IHC scores for pGSK-3β in endometriosis-OCCCa (left) and endometriosis-OEmCa (right). The data shown are means  ± SDs. **d** Correlation between Nodal and pGSK-3β scores in a combination of endometriosis and OCCCa (left) or OEmCa (right)

It was found that the Nodal protein contains a GSK-3β phosphorylation motif (Fig. 3a). To examine an association between Nodal expression and GSK-3β status, Ishikawa cells stably overexpressing wild type Nodal (Ish-NoD#11) or Nodal with a mutation in the putative GSK-3β phosphorylation motif (2TA) (Ish-NoD-2TA#11) were established, since these cells had low endogenous Nodal expression. Treatment of Ish-NoD#11 cells with LiCl, an inhibitor of GSK-3β, with or without MG132, a proteasome inhibitor, resulted in increased Nodal expression as compared to that without the pretreatment, in contrast to the minor effects in Ish-NoD2TA#11 cells (Fig. 3a).

In clinical samples, pGSK-3β immunoreactivity showed cytoplasmic and/or nuclear staining in epithelial components that was frequently associated with the Nodal status (Fig. 3b). Average IHC scores for pGSK-3β showed stepwise decreases from E1 and E2 through to OCCCa, while the scores were extremely low in OEmCa and E2 as compared to that of E1 lesions (Fig. 3c). There was a positive correlation between pGSK-3β scores and Nodal scores in both endometriosis-OCCCa and endometriosis-OEmCa lesions (Fig. 3d and Table 2). These findings suggest that Nodal expression is closely associated with changes in GSK-3β activity in endometriosis-carcinoma lesions, particularly in OCCCa.

**Table 2:** Correlations among Nodal and its related molecules in OCCCas and OEmCas

	OCCCa							OEmCa						
	Nodal	pSmad2	XIAP	bcl2	bax	pGSK-3β	Ki-67	Nodal	pSmad2	XIAP	bcl2	bax	pGSK-3β	Ki-67
	*r* (p)	*r* (p)	*r* (p)	*r* (p)	*r* (p)	*r* (p)	*r* (p)	*r* (p)	*r* (p)	*r* (p)	*r *(p)	*r* (p)	*r* (p)	*r* (p)
pSmad	0.48	*	*	*	*	*	*	0.25	*	*	*	*	*	*
	(0.0003)							(0.18)						
XIAP	0.61	0.46	*	*	*	*	*	0.43	0.1	*	*	*	*	*
	(< 0.0001)	(0.0006)						(0.02)	(0.57)					
bcl2	0.14	0.4	0.2	*	*	*	*	0.18	−0.01	−0.29	*	*	*	*
	(0.28)	(0.002)	(0.14)					(0.3)	(0.93)	(0.13)				
bax	− 0.06	0.14	− 0.19	0.2	*	*	*	0.4	0.08	0.16	0.26	*	*	*
	(0.64)	(0.29)	(0.49)	(0.14)				(0.03)	(0.65)	(0.39)	(0.15)			
pGSK-3β	0.4	0.47	0.4	0.4	0.4	*	*	0.46	0.5	0.61	0.35	0.7	*	*
	(0.003)	(0.0005)	(0.003)	(0.002)	(0.003)			(0.01)	(0.007)	(0.001)	(0.05)	(0.0001)		
Ki-67	0.07	0.22	0.11	0.02	0.1	0.46	*	−0.13	0.19	0.36	−0.28	0.36	0.6	*
	(0.58)	(0.1)	(0.8)	(0.89)	(0.46)	(0.0006)		(0.49)	(0.3)	(0.05)	(0.12)	(0.05)	(0.001)	
Apoptosis	0.5	0.32	0.25	0.09	0.05	0.24	0.1	0.11	−0.07	−0.19	0.32	0.43	0.32	−0.17
	(0.0002)	(0.02)	(0.06)	(0.5)	(0.73)	(0.07)	(0.47)	(0.59)	(0.7)	(0.3)	(0.08)	(0.02)	(0.08)	(0.93)

*r*, Spearman’s correlation coefficient; *, not examined

### Relationship of nodal expression with susceptibility to apoptosis in OCCCa

**Fig. 4:**
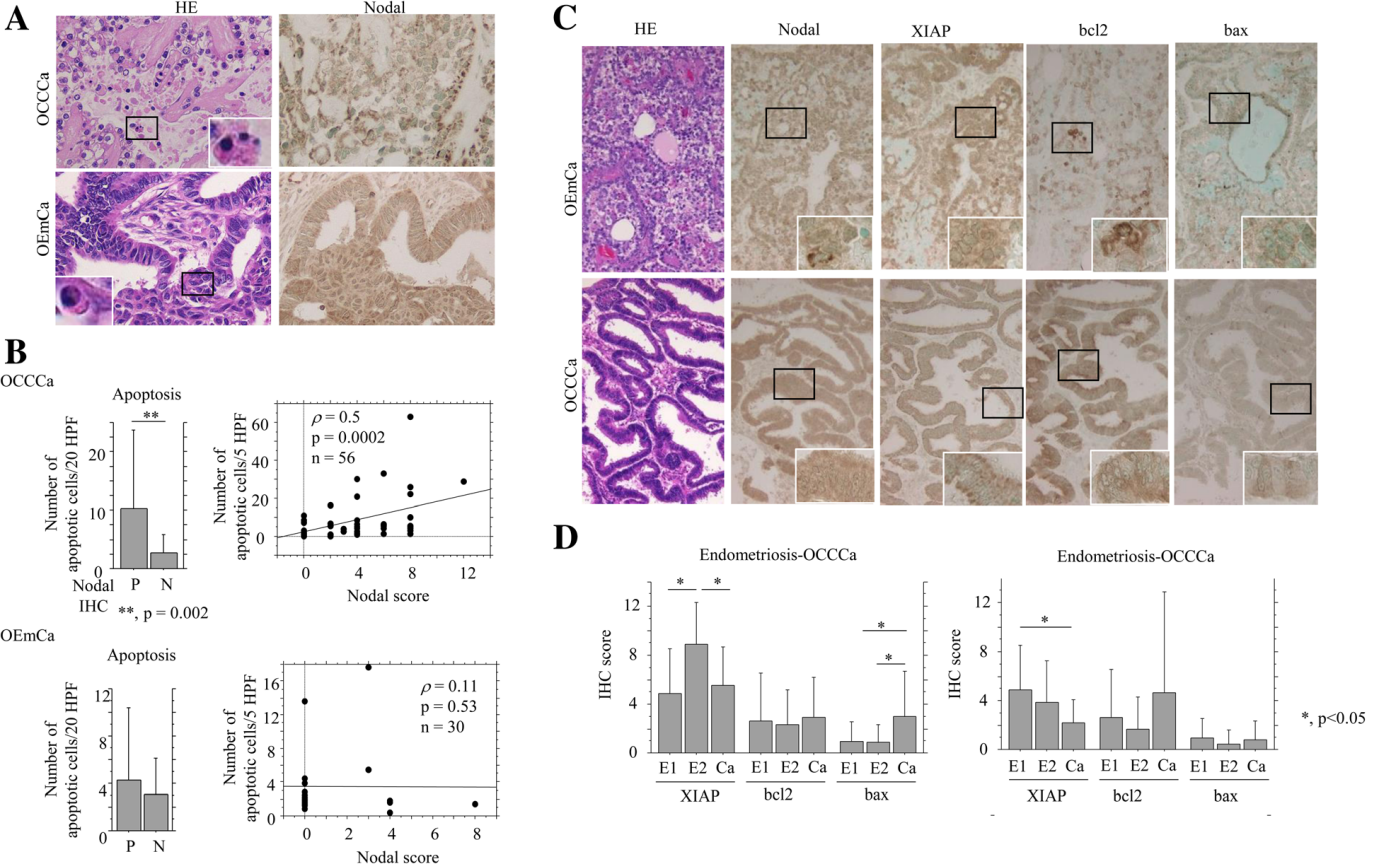
Association between overexpression of Nodal and susceptibility to apoptosis in OCCCa and OEmCa. **a** Staining by hematoxylin and eosin (HE) and IHC for Nodal in OCCCa and OEmCa. Insets show the magnified views of the boxed areas, demonstrating typical apoptotic cells. Original magnification, × 200, × 400 (inset). **b** Left: number of apoptotic cells detected in HE sections of OCCCa (upper) and OEmCa (lower) between positive (P) (score≧ 1) and negative (N) Nodal categories (score = 0). The data shown are means ± SDs. Right: correlation between Nodal score and number of apoptotic cells in OCCCa (upper) and OEmCa (lower). **c** Staining by hematoxylin and eosin (HE) and IHC for the indicated proteins in OCCCa and OEmCa. Insets show the magnified views of the boxed areas. Original magnification, × 200, × 400 (inset). **d** IHC scores for the indicated proteins in a combination of endometriosis and OCCCa (left) or OEmCa (right). E1, endometriosis without carcinoma; E2, endometriosis with carcinoma; Ca, carcinoma. The data shown are means ± SDs

To examine the role of Nodal on cellular phenotypes, we first investigated whether Nodal is associated with apoptosis in OCCCa and OEmCa. In clinical samples, apoptotic cells were readily detected in HE-stained sections of both OCCCa and OEmCa lesions on the basis of characteristic features (Fig. 4a): these results were similar to those obtained with the TUNEL assay (data not shown) as described previously [22]. In contrast, apoptotic cells were difficult to be detected in endometriotic lesions because of frequent inflammatory cell infiltrations. As shown in Fig. 4b, the average of apoptotic values were significantly higher in Nodal-positive (score ≧ 1) as compared to Nodal-negative (score = 0) OCCCas, but not OEmCas, in line with the results showing a positive correlation between the two values (Fig. 4b).

Next, we examined associations of Nodal expression with apoptosis-related molecules including XIAP, Bcl2, and Bax. Immunoreactivities for XIAP, Bcl2, and Bax were mainly detected in cytoplasmic compartments and was heterogeneously distributed within tumor lesions (Fig. 4c). The bax scores were significantly increased in OCCCa as compared to E1 and E2 lesions, while XIAP scores showed stepwise decreases from E1 and E2 through to OEmCa (Fig. 4d). The XIAP score was positively correlated with Nodal and pSmad2 scores in OCCCa and OEmCa, while Bax and Bcl2 scores showed no associations with Nodal, pSmad2, and XIAP scores, with the exception of some cases (Table 2).

**Fig. 5:**
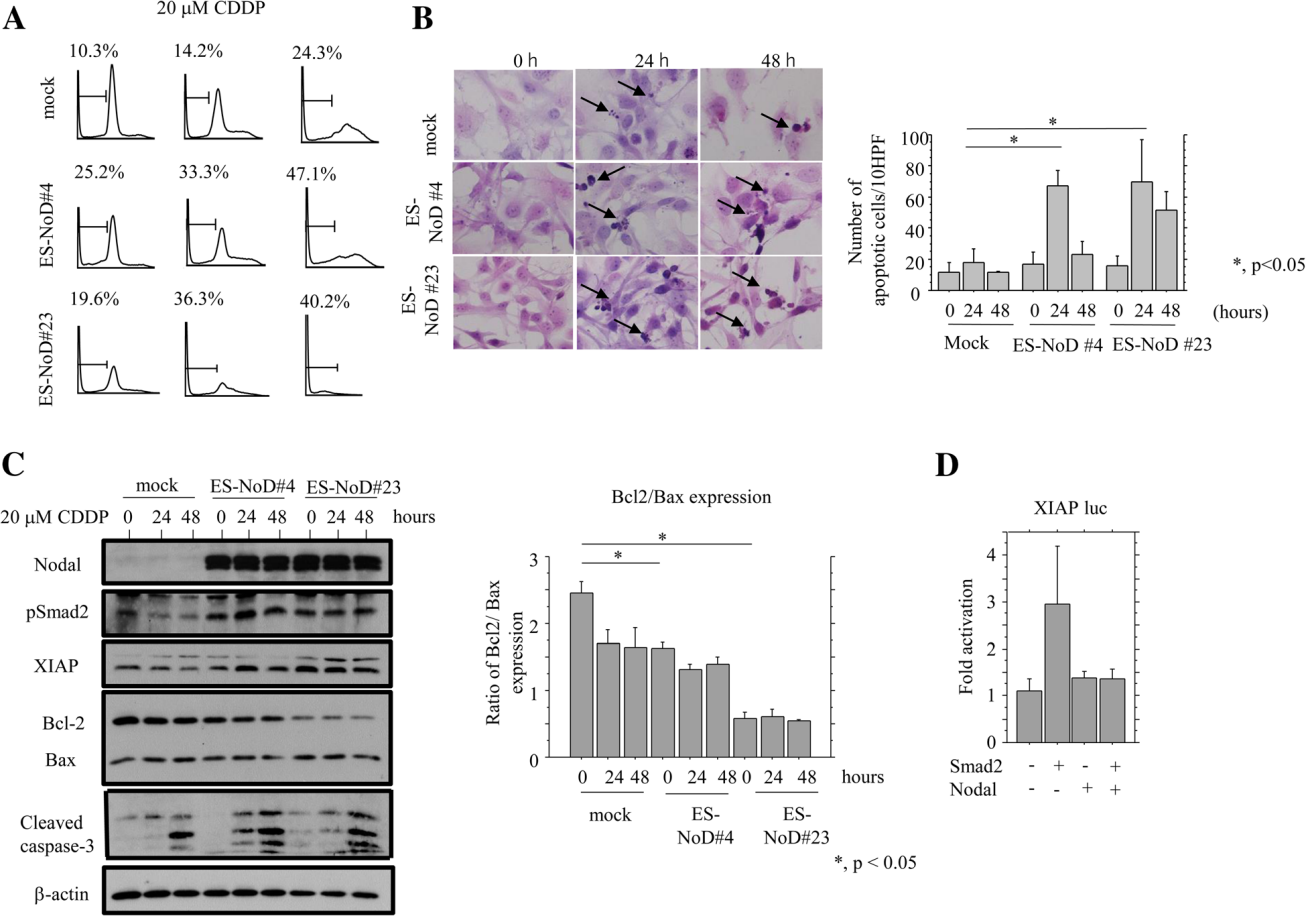
Overexpression of Nodal enhances susceptibility to apoptosis in OCCCa cells. **a** After treatment of the stable Nodal-expressing ES-NoD and mock cells with 20 μM CDDP for the times indicated, cells undergoing apoptosis (sub-G1) were detected by flow cytometry. This experiment was performed in triplicate using independent samples. **b** Left: after treatment with 20 μM CDDP, the stable Nodal-expressing ES-NoD and mock cells undergoing apoptosis (indicated by arrows) were detected by TUNEL assay. Original magnification, × 400. Right: number of apoptotic cells per 10 high power fields (HPFs) detected by TUNEL assay for the times shown. **c** Left: western blot analysis of the indicated proteins in the stable Nodal-expressing ES-NoD and mock cells after 20 μM CDDP treatment for the times shown. Right: values of endogenous Bcl2 relative to Bax protein were calculated by normalization to β-actin in the stable Nodal-expressing ES-NoD and mock cells after 20 μM CDDP treatment for the times shown. **d** Ishikawa cells were transfected with XIAP reporter constructs, together with cotransfection of Smad2 and/or Nodal. Relative activity was determined based on arbitrary light units of luciferase activity normalized to pRL-TK activity. The activities of the reporter plus the effector relative to that of the reporter plus empty vector are shown as means ± SDs. The experiment was performed in duplicate

To further examine a direct association between Nodal expression and apoptosis, cell lines stably overexpressing Nodal were also established using ES-2 cells, which showed a weak endogenous Nodal expression as compared to that of OVISE and TOV-21G cells (Additional file 1: Figure S1B). Treatment of the stable Nodal-expressing ES-2 cells (ES-NoD#4 and #23) with CDDP resulted in an increased proportion of cells in sub-G1 (apoptotic cells) phase along with a decrease in the G1 fraction (Fig. 5a), in line with the results of the TUNEL assay (Fig. 5b). The expression of pSmad2, XIAP, and cleaved caspase-3 were also increased in the stable Nodal-expressing cells than in mock cells, in contrast to a decreased Bcl2/Bax ratio, particularly in ES-NoD#23 cells (Fig. 5c). The *XIAP* promoter was activated three-fold by Smad2 transfection, but this effect was abrogated by cotransfection of Nodal (Fig. 5d). Similar findings were also observed in Ish-NoD cells stably overexpressing Nodal when they were treated with Dox, with the exception of the Bcl2/Bax ratio (Additional file 2: Figure S2). In addition, treatment of Ish-NoD cells with TGF-β1 resulted in a decreased Bcl2/Bax ratio, along with increased expression of pSmad2 and XIAP (Additional file 3: Figure S3). These findings suggest that overexpression of Nodal is closely linked to susceptibility to apoptosis through alteration in expression of pSmad2 and XIAP, and ratio of Bcl2/Bax in OCCCa.

### Relationship of nodal expression with cell proliferation in endometriosis- OCCCa lesions

**Fig. 6:**
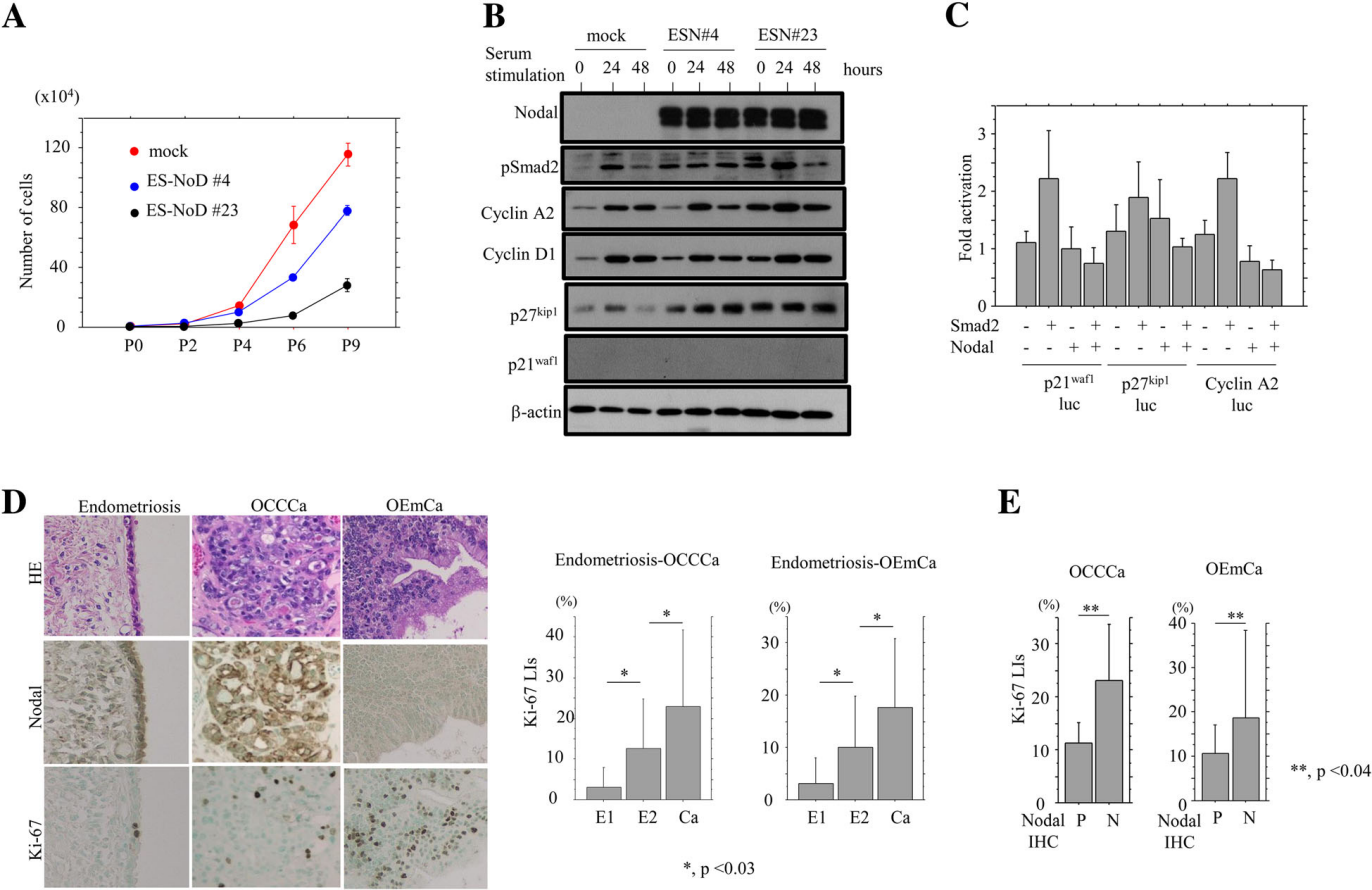
Association between overexpression of Nodal and cell proliferation in endometriosis, OCCCa, and OEmCa. **a** Two independent stable Nodal-expressing ES-NoD cell lines and mock cells were seeded at low density. The cell numbers are presented as mean ± SD. P0, P2, P4, P6, and P9 indicate 0, 2, 4, 6, and 9 days after cell passage, respectively. **b** Western blot analysis for the indicated proteins in the stable Nodal-expressing ES-NoD and mock cells for the times shown following restimulation with 10% serum after serum starvation for 6 h. **c** Ishikawa cells were transfected with p21^waf1^, p27^kip1^, and Cyclin A2 reporter constructs, together with cotransfection of Smad2 and Nodal. Relative activity was determined based on arbitrary light units of luciferase activity normalized to pRL-TK activity. The activities of the reporter plus the effector relative to that of the reporter plus empty vector are shown as means ± SDs. The experiment was performed in duplicate. **d** Left: staining by hematoxylin and eosin (HE) and IHC for Nodal and Ki-67 in endometriosis, OCCCa, and OEmCa. Original magnification, × 200. Middle and right: Ki-67 labeling indices in endometriosis-OCCCa (middle) and endometriosis-OEmCa (right). The data shown are means ± SDs. E1, endometriosis without carcinoma; E2, endometriosis with carcinoma; Ca, carcinoma. **e** Ki-67 labeling indices in OCCCa (left) and OEmCa (right) between Nodal positive (P) (score ≧ 1) and negative (N) categories (score = 0). The data shown are means ± SDs

Both ES-NoD and Ish-NoD cells that stably overexpressed Nodal had a low proliferation rate, particularity in the exponential growth phase (Fig. 6a and Additional file 4: Figure S4A). To further examine alterations in expression of several cell cycle-related molecules during cell growth, the Nodal-overexpressing cells were rendered quiescent by serum starvation and then were serum-stimulated. At 24 and 48 h after stimulation, increased expression of cyclin A2, p27^Kip1^, and p21^waf1^, but not Cyclin D1, were observed in both stable Nodal-overexpressing cells as compared to the mock cells (Fig. 6b and Additional file 4: S4B). Transfection with Smad2 led to increased promoter activity of *p21*^*waf1*^ and *p27*^*kip1*^, as well as *Cyclin A2* genes, while these effects were abrogated by cotransfection of Nodal (Fig. 6c). In clinical samples, average Ki-67 LIs showed stepwise increases from E1 and E2 through to carcinoma lesions of both OCCCa and OEmCa (Fig. 6d). In addition, the Ki-67 LI values were significantly lower in Nodal-positive (score ≧ 1) as compared to Nodal-negative (score = 0) samples of both OEmCa and OCCCa (Fig. 6e). These findings suggest that Nodal participates in modulation of the cell proliferation rate through regulation of expression of several cell cycle-related molecules.

Finally, Nodal and several cell-kinetics-related factors investigated were not associated with clinical stages in both OCCCa and OEmCa, with the exception of some cases (Additional file 5: Figure S5).

## Discussion

The present study clearly provided evidence that high Nodal mRNA and protein expression were found to be very tightly linked with endometriosis- OCCCa lesions as compared to non-OCCCa. Treatment of cells with TGF-β1 resulted in transcriptional activation of the *Nodal* gene, along with increased pSmad2 expression. Moreover, Nodal expression was significantly higher in the epithelial components of endometriotic cysts as compared to that of OCCCa, again with a positive correlation with pSmad2 expression. A similar finding was also observed in left-right determination factor (LEFTY), which is a member of the TGF-β superfamily. Specifically, the cytoplasmic fraction of LEFTY can repress TGF-β signaling activity by inhibiting Smad2 phosphorylation after activation of the TGF-β receptor [23, 25]. Interestingly, characterization of the transcriptional regulatory mechanisms of *Nodal* and *LEFTY* has revealed the presence of complex regulatory loops involving the two gene, in which Nodal upregulates the expression of its own gene as well as that of *LEFTY* [26]. Given our previous data that show LEFTY is an excellent OCCCa-specific molecular marker [22], it is possible that Nodal may also be a specific biomarker for OCCCa. In addition, our findings showing high TGF-β1 mRNA expression in most OECa samples, as well as endometriotic lesions allow us to speculate that the presence of anti-Smad proteins (Smad6 and Smad7) and repressors (Sno N and Ski) for TGF-β1 responsive genes may be in part due to decreased Nodal expression in the tumors, particularly in non-OCCCas. The status of cell surface receptors including types I and II for TGF-β signal transduction may also be important, since epithelial Mv1Lu cells lacking these receptors were completely unresponsive to TGF-β-induced gene expression [27].

We also found that Nodal expression was posttranslationally regulated through alteration in GSK-3β activity in endometriosis-OCCCa lesions. We found that Nodal protein contains one putative GSK-3β phosphorylation motif, which suggests a functional relationship between these two proteins. Further, inhibition of GSK-3β by LiCl resulted in increased Nodal expression, while these effects were abrogated by mutations in the putative GSK-3β phosphorylation motif in the protein. Similar findings were also observed for the LEFTY protein. In addition, Nodal expression was positively correlated to pGSK-3β status in endometriosis-OCCCa lesions. Given our previous data showing that TGF-β signaling leads to functional inhibition of GSK-3β activity through pAkt activation [23], it appears that Nodal expression may be regulated by both Smad- and non-Smad-dependent pathways in response to TGF-β action in the endometriosis-OCCCa lesions.

In line with several reports showing that Nodal exerts proapoptotic effects in various cell lineages [28, 29], its expression was significantly associated with a higher number of apoptotic cells in OCCCa tissues, despite a lack of direct association with Bcl2 and Bax statuses. In addition, treatment of cells stably overexpressing Nodal with CDDP or Dox resulted in increased apoptotic cells as compared to that of mock cells. These treated cells also showed an increase in expression of pSmad2 and cleaved caspase-3 and decreased ratio of Bcl2/Bax, which induced the release of cytochrome *C*, activation of caspase-3 and subsequent apoptosis. Unexpectedly, expression of XIAP, an inhibitor for apoptosis, was also increased during the process. *XIAP* promoter activation mediated by Smad2 was abrogated by cotransfection of Nodal, despite a positive correlation between Nodal and XIAP expression in OCCCa tissues. These anomalous results may be due to the existence of negative feedback mechanisms among Smad2, Nodal, and XIAP.

Our data also showed that treatment of Ish-NoD stable cells with TGF-β1 induced a decreased ratio of Bcl2/Bax, along with an increased expression of pSmad2 and XIAP. In general, ectopic expression of Smads has been shown to enhance TGF-β-induced apoptosis in certain cells [30–32]. Together with the evidence showing that Nodal is functionally active in inducing downstream Smad2 phosphorylation, which in turn regulates the expression of Bax and Bcl2 [33, 34], it is possible that overexpression of Nodal may be indirectly linked with disruption of the mitochondrial membrane potential through alteration in Smad2-mediated expression of XIAP, Bcl2, and Bax. This finding is supported by a report showing that Nodal-activin receptor-like kinase (ALK) 7-induced apoptosis is induced at least in part by two Smad-dependent pathways, Bcl2/Bax and XIAP, in OECa cell lines [35].

Another interesting finding in this study was that overexpression of Nodal led to the inhibition of cell proliferation, along with increased expression of p27^kip1^ and/or p21^waf1^, as well as Cyclin A2, probably through their posttranscriptional modulation, since Smad2-mediated activation of these promoters was abrogated by cotransfection of Nodal. In clinical samples, Ki-67 LI values showed stepwise increases from endometriosis-OCCCa lesions, in contrast to decreased Nodal expression. Moreover, significantly decreased Ki-67 LIs were observed in Nodal-positive OCCCa as compared to the negative cases. TGF-β inhibits proliferation in a cell type-dependent manner by arresting cells in the G1 phase of the cell cycle [36]. Therefore, it appears that Nodal may serve as an inhibitor of cell proliferation through alteration in expression of cell cycle-related molecules in response to TGF-β1 signaling. In human trophoblast cells, the Nodal-ALK7 pathway inhibits cell proliferation by inducing G_1_ cell cycle arrest mediated in part by the p27^kip1^-Cyclin E/Cdk2 pathway [37].

## Conclusion

**Fig. 7:**
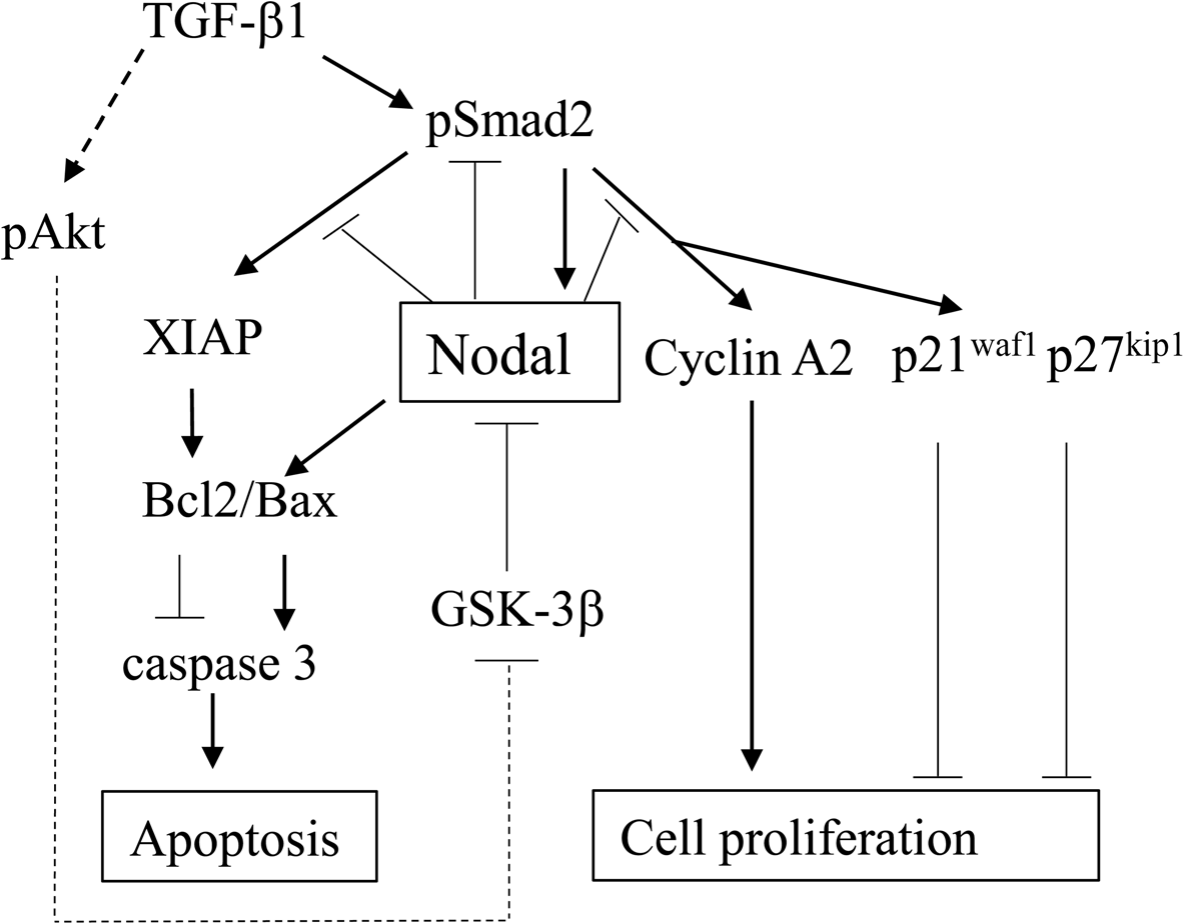
Schematic representation of association of Nodal with susceptibility to apoptosis and cell proliferation in endometriosis- OCCCa

Together, our observations suggest a model for the functional role of Nodal in ovarian endometriosis-carcinoma, particularly in OCCCa (Fig. 7). Upregulation of Nodal expression mediated by both activation of TGF-β1/Smad signaling and inactivation of GSK-3β results in an enhancement of apoptotic features through decreased Bcl2/Bax ratio and an inhibition of cell proliferation due to changes in expression of several cell cycle-related markers. Thus, Nodal is a multifunctional cytokine involved that plays a key role in the biology of ovarian endometriosis-carcinoma lesions. In line with the evidence for the clinical potential of targeting Nodal in several human malignancies [38], it is possible that Nodal could also represent a candidate biomarker for OCCCa progression.

## Additional files

Additional file 1: **Figure S1.** Responsiveness for TGF-β1 and endogenous Nodal status in the cell line investigated. (A) Western blot for the indicated proteins after treatment of ES-2, OVISE, TOV-21G, and Ishikawa cells with 2 ng/mL TGF-β1 for the times indicated. (B) Western blot for the indicated proteins in ES-2, OVISE, and TOV-21G cells. (TIF 1163 kb)

Additional file 2: **Figure S2**. Overexpression of Nodal enhances susceptibility to apoptosis in OEmCa cells. (A) After treatment of the stable Nodal-expressing Ish-NoD and mock cells with 1 μg/mL Dox for the times indicated, cells undergoing apoptosis (sub-G1) were detected by flow cytometry. This experiment was performed in triplicate using independent samples. (B) Upper: after treatment with 1 μg/mL Dox, the stable Nodal-expressing Ish-NoD and mock cells undergoing apoptosis (indicated by arrows) were detected by TUNEL assay. Original magnification, × 400. Lower: number of apoptotic cells per 10 high power fields (HPFs) detected by TUNEL assay for the times shown. (C) Left: western blot analysis of the indicated proteins in the stable Nodal-expressing Ish-NoD and mock cells after 1 μg/mL Dox treatment for the times shown. Right: values of endogenous Bcl2 relative to Bax protein were calculated by normalization to β-actin in the stable Nodal-expressing Ish-NoD and mock cells after 1 μg/mL Dox treatment for the times shown. (TIF 3448 kb)

Additional file 3: **Figure S3.** Association between TGF-β1 with Bcl2/Bax status in the stable Nodal-expressing Ish-NoD cells. Left: western blot assay for the indicated proteins after treatment of Ishikawa cells with 2 ng/mL TGF-β1 for the times indicated. Right: values of endogenous bcl2 relative to bax protein were calculated by normalization to β-actin in Ishikawa cells after 2 ng/mL TGF-β1 for the times indicated. (TIF 1720 kb)

Additional file 4: **Figure S4.** Association between overexpression of Nodal and cell proliferation in OEmCa. (A) Two independent stable Nodal-expressing Ish-NoD cell lines and mock cells were seeded at low density. The cell numbers are presented as mean ± SD. P0, P2, P4, P6, and P9 indicate 0, 2, 4, 6, and 9 days after cell passage, respectively. (B) Western blot analysis for the indicated proteins in the stable Nodal-expressing Ish-NoD and mock cells for the times shown following restimulation with 10% serum after serum starvation for 6 h. (TIF 1576 kb)

Additional file 5: **Figure S5.** Association of clinical stages with the indicated factors in OCCCa (upper) and OEmCa (lower). (TIF 758 kb)

## Abbreviations

GSK-3β: Glycogen synthase kinase 3β
IHC: Immunohistochemistry
ISH: In situ hybridization
LEFTY: Left-right determination factor
OCCCa: Ovarian clear cell carcinoma
OECa: Ovarian epithelial carcinoma
OEmCa: Ovarian endometrioid carcinoma
TGF-β: Transforming growth factor β
XIAP: X-linked inhibitor of apoptosis

## References

[1] Anglesio MS, Carey MS, Kobel M, Mackay H, Huntsman DG (2011). Vancouver Ovarian Clear Cell Symposium Speaker. Clear cell carcinoma of the ovary: a report from the first ovarian clear Cee symposium, June 24 th, 2010. Gynecol Oncol.

[2] Skirnisdottir I, Seidal T, Karlsson MG, Sorbe B (2005). Clinical and biological characteristics of clear cell carcinoma of the ovary in FIGO stages I-II. Int J Oncol.

[3] Sugiyama T, Kamura T, Kigawa J, Terakawa N, Kikuchi Y, Kita T, Suzuki M, Sato I, Taguchi K (2000). Clinical characteristics of clear cell carcinoma of the ovary: a distinct histologic type with poor prognosis and resistance to platinum-based chemotherapy. Cancer..

[4] Chan JK, Teoh D, Hu JM, Shin JY, Osann K, Kapp DS (2008). Do clear cell ovarian carcinoma have poorer prognosis compared to other epithelial types? A study of 1411 clear cell ovarian cancers. Gynecol Oncol.

[5] Itamochi H, Kigawa J, Toyota N (2008). Mechanisms of chemoresistance and poor prognosis in ovarian clear cell carcinoma. Cancer Sci.

[6] Olive DI (2003). Medical therapy of endometriosis. Semin Reprod Med.

[7] Massaguie J (2008). TGFbeta in Cancer. Cell..

[8] Reiss M (1999). TGF-β and cancer. Microbes Infect.

[9] Yamaguchi K, Mandai M, Toyokuni S, Mamanishi J, Higuchi T, Takakura K, Fujii S (2008). Contents of endometriotic cysts, especially the high concentration of free iron, are a possible cause of carcinogenesis in the cysts through the iron-induced persistent oxidative stress. Clin Cancer Res.

[10] Jaowlew SB (2006). Transforming growth factor-β in cancer and metastasis. Cancer Metastasis Rev.

[11] Ikushima H, Miyazono K (2010). TGFbeta signaling: a complex web in cancer progression. Nat Rev Cancer.

[12] Joshi A, Cao D (2010). TGF-beta signaling: tumor microenvironment and tumor progression: the butterfly effect. Front Biosci.

[13] Moustakas A, Souchelnytskyl S, Heldin C-H (2001). Smad regulation in TGF-β signal transduction. J Cell Sci.

[14] Brennan J, Norris DP, Robertson EJ (2002). Nodal activity in the node governs left-right asymmetry. Gen Dev.

[15] Takenaga M, Fukumoto M, Hori Y (2007). Regulated nodal signaling promotes differentiation of the definitive endoderm and mesoderm from ES cells. J Cell Sci.

[16] Eimon PM, Harland RM (2002). Effects of heterodimerization and proteolytic processing on derriere and nodal activity: implications for mesoderm induction in Xenopus. Development.

[17] Schier AF (2003). Nodal signaling in vertebrate development. Annu Rev Cell Dev Biol.

[18] Topczewska JM, Postovit L-M, Margaryan NV, Sam A, Hess AR, Wheaton WW, Nickoloff BJ, Topczewski J, Hendrix MJC (2006). Embryonic and tumorigenic pathways converge via nodal signaling: role in melanoma aggressiveness. Nat Med.

[19] Xu G, Zhong Y, Munir S, Yang BB, Tsang BK, Peng C (2004). Nodal induces apoptosis and inhibits proliferation in human epithelial ovarian cancer cells via activin receptor-like kinase 7. J Clin Endocrinol Metab.

[20] Longacre TA, Wells M, Bell DA, Malpica A, Prat J, Ronnet BM, Kurman RJ, Carcangiu ML, Herrington CS, Young RH (2014). Tumours of the tumours of the ovary. WHO classification of Tumours of female reproductive organs.

[21] Benedet JL, Bender H, Jones H 3^rd^, Ngan HY, Pecorelli S. FIGO staging classifications and clinical practice guidelines in the management of gynecologic cancers. FIGO committee on gynecologic oncology. Int J Gynaecol Obstet 2000;70:209–262. 11041682

[22] Akiya M, Yamazaki M, Matsumoto T, Kawashima Y, Oguri Y, Kajita S, Kijima D, Chiba R, Yokoi A, Takahashi H, Kodera Y, Saegusa M (2017). Identification of LEFTY as a molecular marker for ovarian clear cell carcinoma. Oncotarget..

[23] Fei W, Kijima D, Hashimoto M, Hashimura M, Oguri Y, Kajita S, Matsumoto T, Yokoi A, Saegusa M (2017). A functional role of LEFTY during progesterone therapy for endometrial carcinoma. Cell Commun Signal.

[24] Kerr JF, Winterford CM, Harmon BV (1994). Apoptosis: its significance in cancer and cancer therapy. Cancer..

[25] Ulloa L, Tabibzadeh S (2001). Lefty inhibits receptor-regulated Smad phosphorylation induced by activated transforming growth factor-beta receptor. J Biol Chem.

[26] Juan H, Hamad H (2001). Role of nodal-lefty regulatory loops in embryonic patterning of vertebrates. Genes Cells.

[27] Feng X-H, Filvaroff EH, Derynck R (1995). Transforming growth factor-β (TGF-β)-induced down-regulation of cyclin a expression requires a functional TGF-β receptor complex. J Biol Chem.

[28] Zhao F, Huang F, Tang M, Li X, Zhang N, Amfilochiadis A, Li Y, Hu R, Jin T, Peng C, Wang Q (2012). Nodal induces apoptosis through activation of the ALK7 signaling pathway in pancreatic INS-1 β-cells. Am J Physiol Endocrinol Metab.

[29] Zhong Y, Xu G, Ye G, Lee D, Modica-Amore J, Peng C (2009). Nodal and activin receptor-like kinase 7 induce apoptosis in human breast cancer cell lines: role of caspase 3. Int Physiol Pathophysiol Pharmacol.

[30] Yanagisawa K, Osada H, Masuda A, Kondo M, Saito T, Yatabe Y, Takagi K, Takahashi T, Takahashi T (1998). Induction of apoptosis by Smad3 and down-regulation of Smad3 expression in response to TGF-β in human normal lung epithelial cells. Oncogene..

[31] Bakkebo M, Huse K, Hilden VI, Smeland EB, Oksvold MP (2010). TGF-β-induced growth inhibition in B-cell lymphoma correlates with Smad1/5 signaling and constitutively active p38 MARK. BMC Immunol.

[32] Bakhshayesh M, Zaker F, Hashemi M, Katebi M, Solaimani M (2012). TGF-β1-mediated apoptosis associated with Smad-dependent mitochondrial bcl-2 expression. Clin Lymphoma Myeloma Leuk.

[33] Lawrence MG, Margaryan NV, Loessner D, Collins A, Kerr KM, Turner M, Seftor EA, Stephens CR, Lai J, BioResource APC, Postovit L-M, Clements JA, Hendrix MJC (2011). Reactivation of embryonic nodal signaling is associated with tumor progression and promotes the growth of prostatic cancer cells. Prostate.

[34] Wang H, Tsang BK (2007). Nodal signaling and apoptosis. Reproduction..

[35] Xu G, Zhou H, Wang Q, Auersperg N, Peng C (2006). Activin receptor-like kinase 7 induces apoptosis through up-regulation of bax and down-regulation of Xiap in normal and malignant ovarian epithelial cell lines. Mol Cancer Res.

[36] Dijke PT, Goumans M-J, Itoh F, Itoh S (2002). Regulation of cell proliferation by Smad protein. J Cell Physiol.

[37] Munir S, Xu G, Wu Y, Yang B, Lala PK, Peng C (2004). Nodal and ALK7 inhibit proliferation and induce apoptosis in human trophoblast cells. J Biol Chem.

[38] Strizzi L, Hardy KM, Kirschmann DA, Ahrlund-Richter L, Hendrix MJC (2012). Nodal expression and detection in cancer: experience and challenges. Cancer Res.

