# Supplementary figures and images for: Nodal induces apoptosis and inhibits proliferation in ovarian endometriosis-clear cell carcinoma lesions
Source: BMC Cancer. 2019 Apr 3;19:308. doi: 10.1186/s12885-019-5539-y (PMC6448249; doi:10.1186/s12885-019-5539-y)

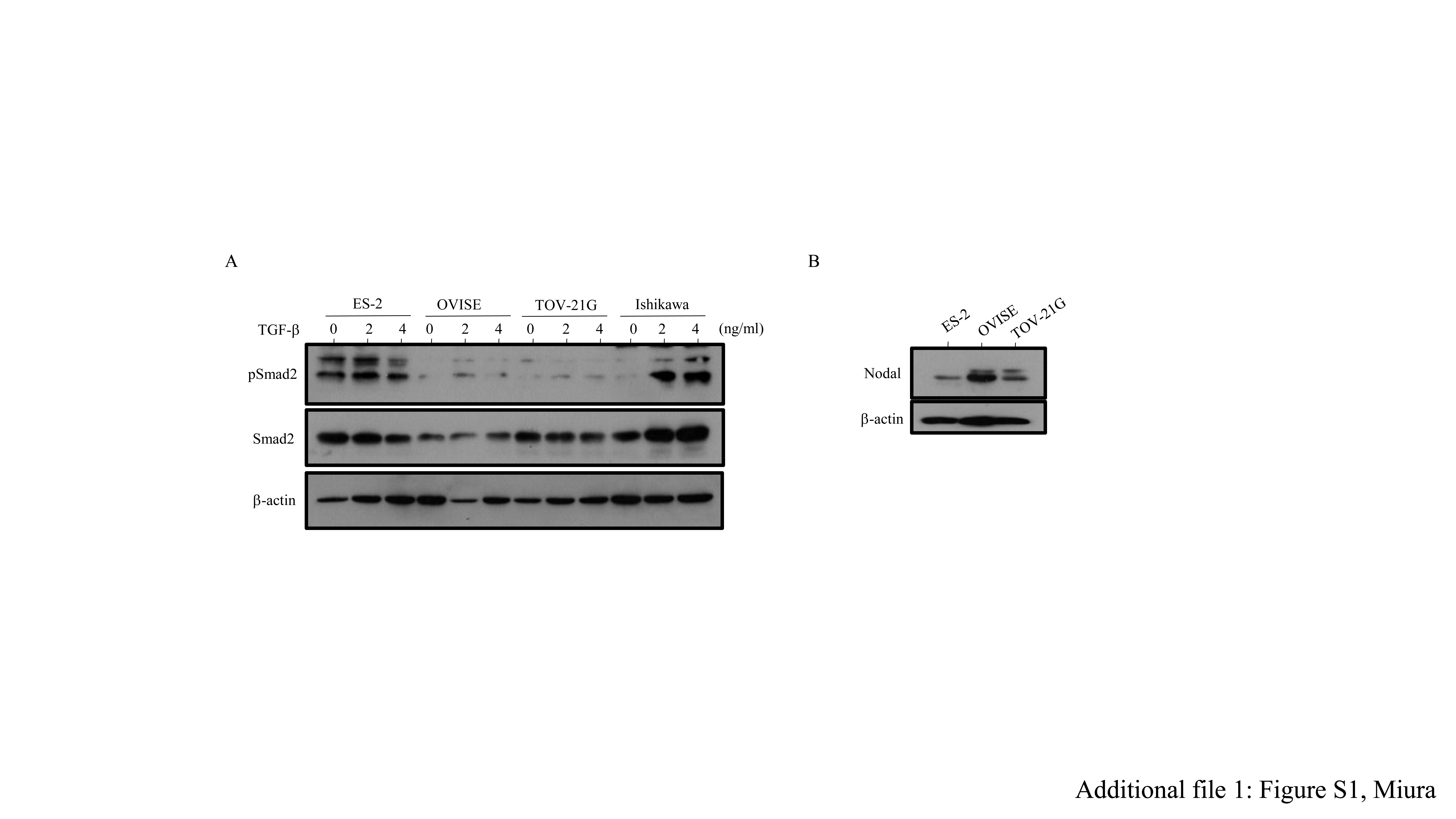

Supplement: Supplementary file 1 — Figure S1. Responsiveness for TGF-β1 and endogenous Nodal status in the cell line investigated. (A) Western blot for the indicated proteins after treatment of ES-2, OVISE, TOV-21G, and Ishikawa cells with 2 ng/mL TGF-β1 for the times indicated. (B) Western blot for the indicated proteins in ES-2, OVISE, and TOV-21G cells. (TIF 1163 kb) [file 12885_2019_5539_MOESM1_ESM.tif]

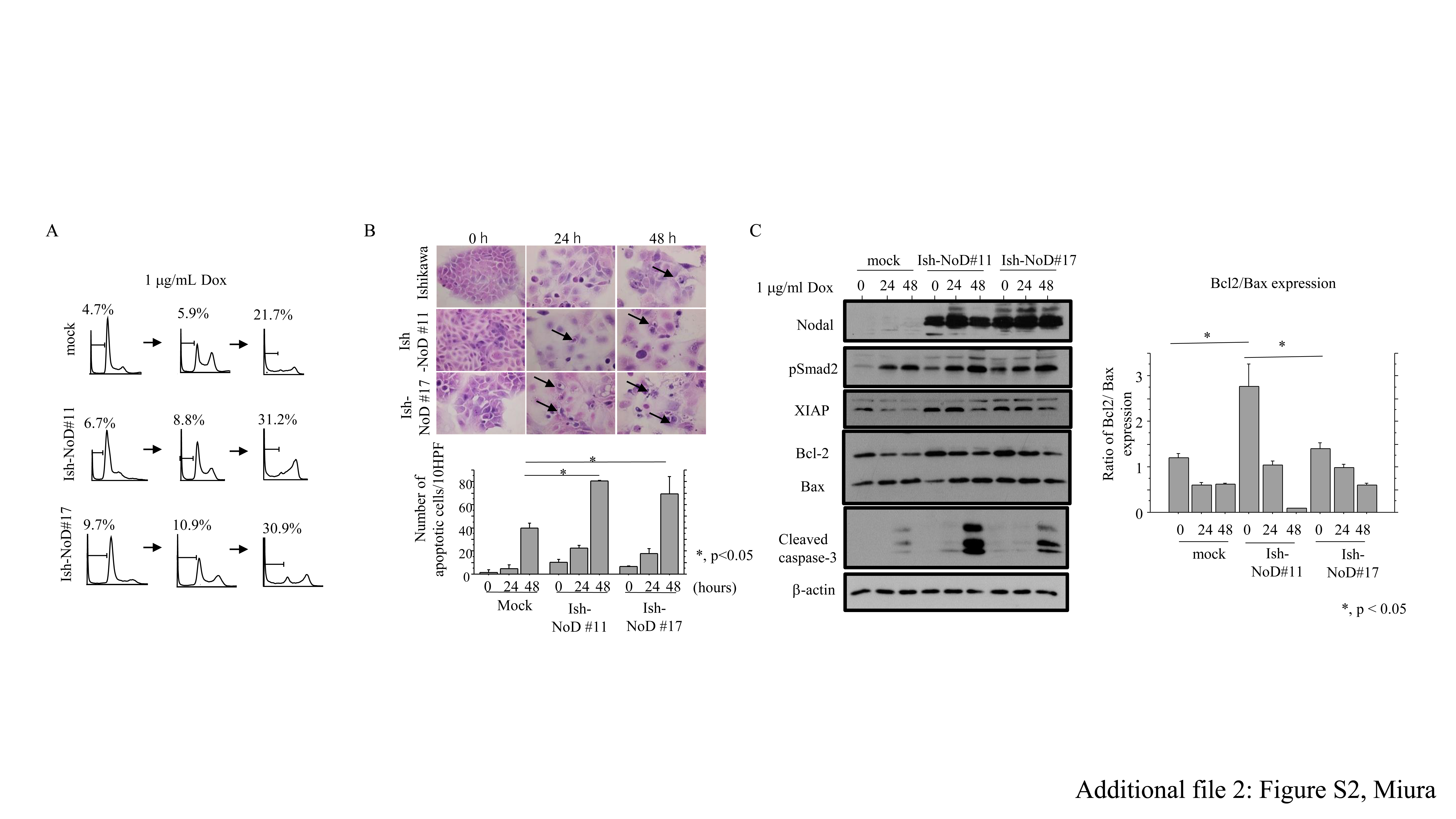

Supplement: Supplementary file 2 — Figure S2. Overexpression of Nodal enhances susceptibility to apoptosis in OEmCa cells. (A) After treatment of the stable Nodal-expressing Ish-NoD and mock cells with 1 μg/mL Dox for the times indicated, cells undergoing apoptosis (sub-G1) were detected by flow cytometry. This experiment was performed in triplicate using independent samples. (B) Upper: after treatment with 1 μg/mL Dox, the stable Nodal-expressing Ish-NoD and mock cells undergoing apoptosis (indicated by arrows) were detected by TUNEL assay. Original magnification, × 400. Lower: number of apoptotic cells per 10 high power fields (HPFs) detected by TUNEL assay for the times shown. (C) Left: western blot analysis of the indicated proteins in the stable Nodal-expressing Ish-NoD and mock cells after 1 μg/mL Dox treatment for the times shown. Right: values of endogenous Bcl2 relative to Bax protein were calculated by normalization to β-actin in the stable Nodal-expressing Ish-NoD and mock cells after 1 μg/mL Dox treatment for the times shown. (TIF 3448 kb) [file 12885_2019_5539_MOESM2_ESM.tif]

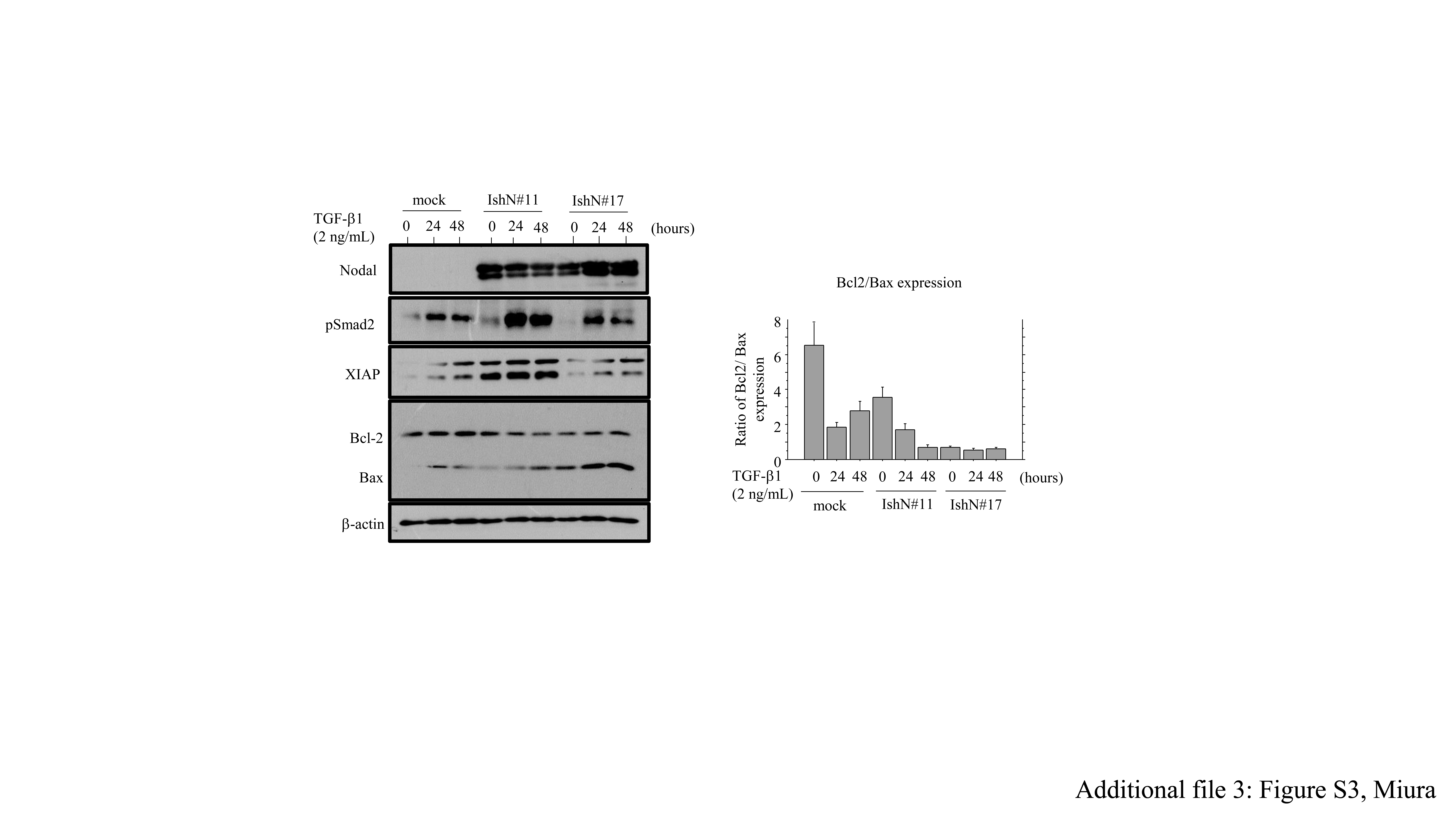

Supplement: Supplementary file 3 — Figure S3. Association between TGF-β1 with Bcl2/Bax status in the stable Nodal-expressing Ish-NoD cells. Left: western blot assay for the indicated proteins after treatment of Ishikawa cells with 2 ng/mL TGF-β1 for the times indicated. Right: values of endogenous bcl2 relative to bax protein were calculated by normalization to β-actin in Ishikawa cells after 2 ng/mL TGF-β1 for the times indicated. (TIF 1720 kb) [file 12885_2019_5539_MOESM3_ESM.tif]

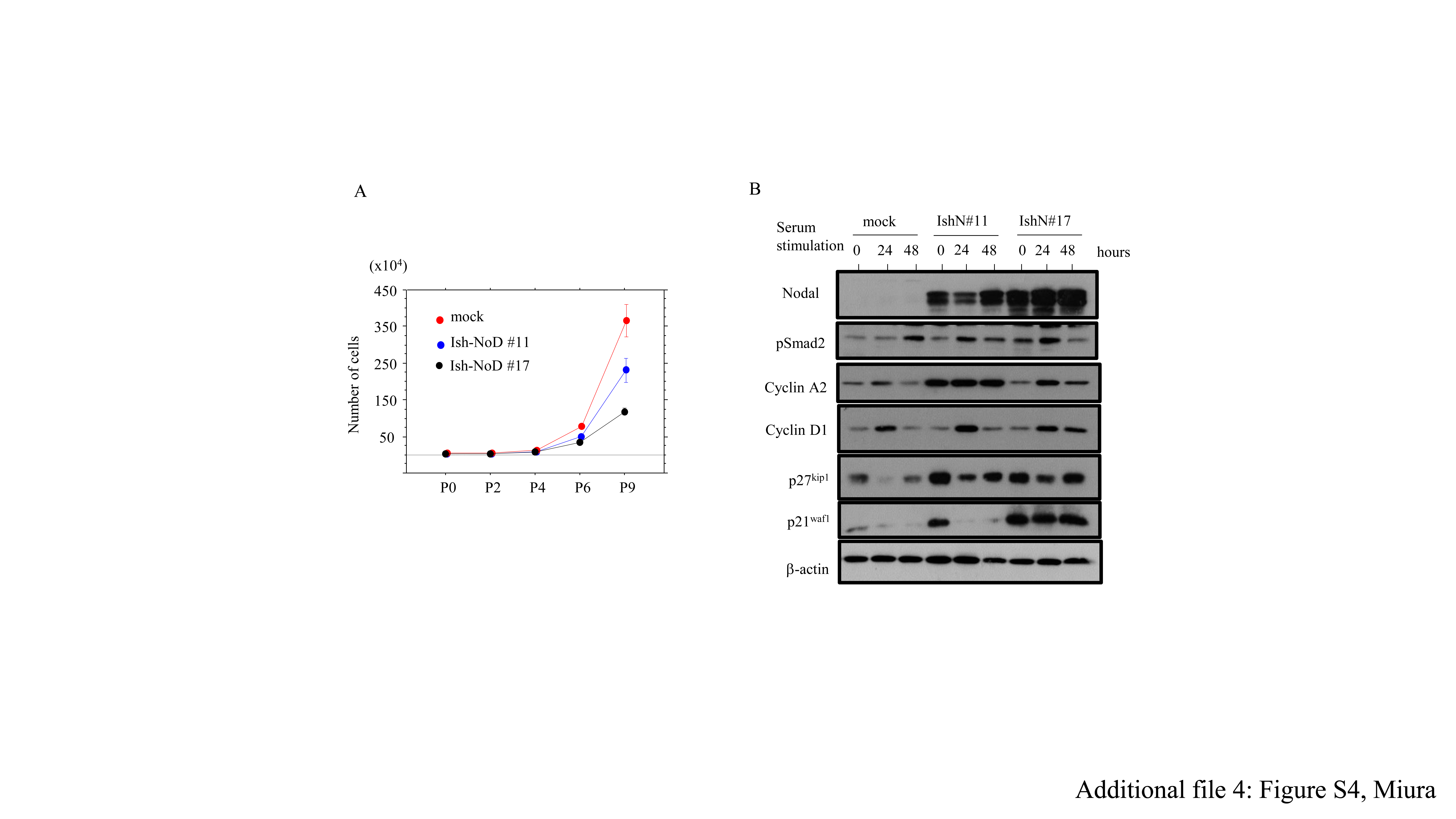

Supplement: Supplementary file 4 — Figure S4. Association between overexpression of Nodal and cell proliferation in OEmCa. (A) Two independent stable Nodal-expressing Ish-NoD cell lines and mock cells were seeded at low density. The cell numbers are presented as mean ± SD. P0, P2, P4, P6, and P9 indicate 0, 2, 4, 6, and 9 days after cell passage, respectively. (B) Western blot analysis for the indicated proteins in the stable Nodal-expressing Ish-NoD and mock cells for the times shown following restimulation with 10% serum after serum starvation for 6 h. (TIF 1576 kb) [file 12885_2019_5539_MOESM4_ESM.tif]

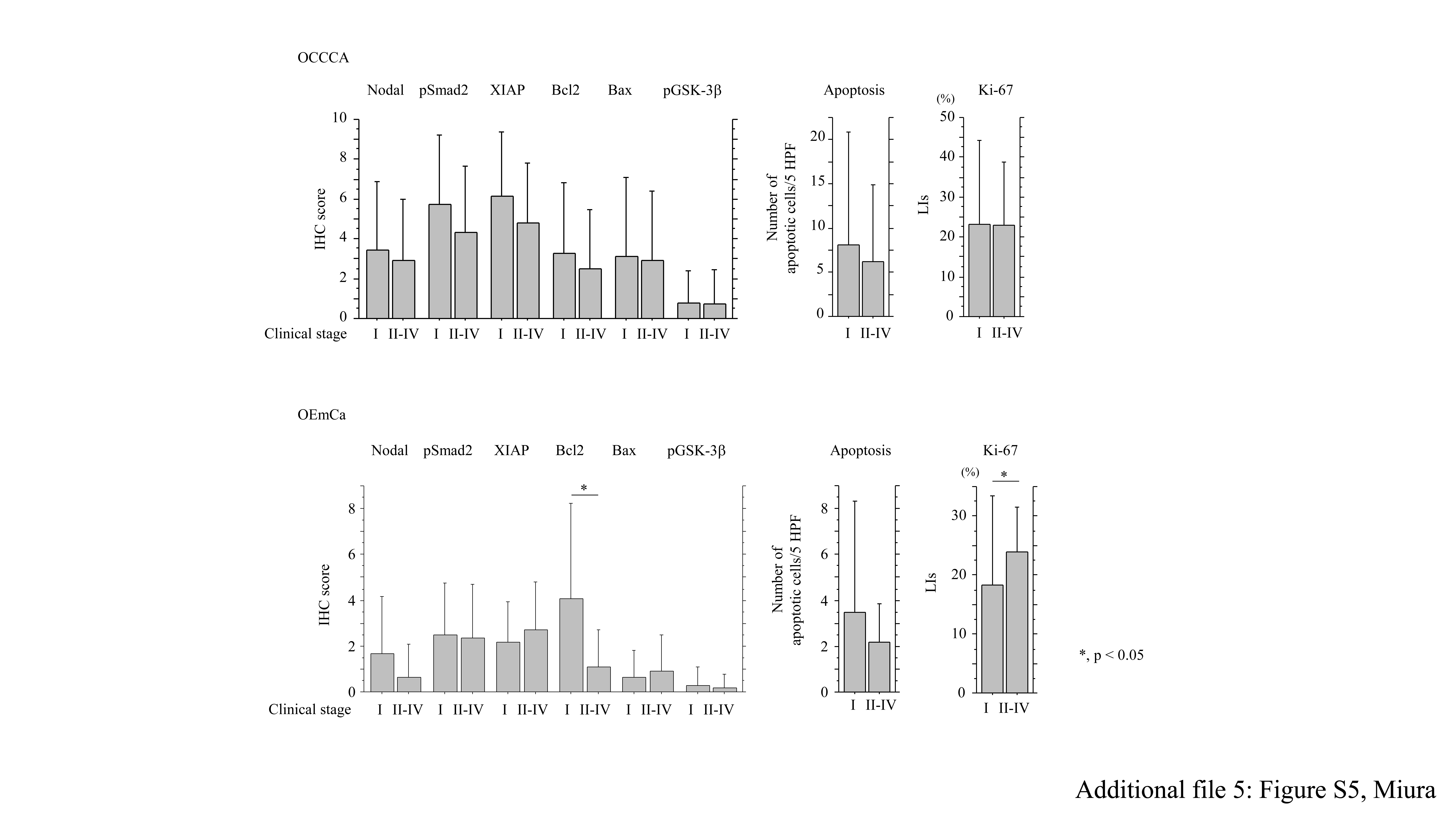

Supplement: Supplementary file 5 — Figure S5. Association of clinical stages with the indicated factors in OCCCa (upper) and OEmCa (lower). (TIF 758 kb) [file 12885_2019_5539_MOESM5_ESM.tif]
